# Discovery of new species of mesoparasitic pennellid (Copepoda: Siphonostomatoida) from the endemic mesopelagic lightfish *Vinciguerria mabahiss* in the Red Sea

**DOI:** 10.1051/parasite/2025038

**Published:** 2025-07-16

**Authors:** Kah Kheng Lim, Carlos Angulo-Preckler, Lotfi J. Rabaoui, Mohammad A. Qurban, Vincent A. Pieribone, Carlos M. Duarte, Daisuke Uyeno

**Affiliations:** 1 Marine Science Program, Biological and Environmental Science and Engineering Division (BESE), King Abdullah University of Science and Technology (KAUST) Thuwal 23955-6900 Kingdom of Saudi Arabia; 2 National Center for Wildlife (NCW), PP2R + WJH Makkah Al Mukarramah Branch Rd, King Abdul Aziz Riyadh 12411 Kingdom of Saudi Arabia; 3 OceanX 37 West 39th St, 8th Floor New York NY 10018 USA; 4 Graduate School of Science and Engineering, Kagoshima University 1-21-35 Korimoto Kagoshima City Kagoshima 890-0065 Japan

**Keywords:** Phosichthyidae, Mesoparasite, *Cardiodectes*, Western Indian Ocean

## Abstract

A new species of the genus *Cardiodectes* Wilson, 1917 (Siphonostomatoida: Pennellidae), *Cardiodectes tofaili* n. sp., is described based on 13 adult females from ten specimens of the endemic lightfish *Vinciguerria mabahiss* (Stomiiformes: Phosichthyidae). These hosts were inadvertently captured by a remotely operated vehicle at depths of 454–645 m in the pelagic waters of the Saudi Arabian Red Sea. The new species is placed under the “*rubosus*” group, characterized by possession of a trunk without a discrete abdomen. It is distinguished from its 12 congeners within this group by having a short neck region with a distinct fourth pedigerous somite, and a trunk that is *ca.* 5 times longer than wide. Phylogenetic analysis based on concatenated *18S* + *28S* rDNA sequences supports the distinctiveness of the new species. This species is endemic to the Red Sea, representing the first recorded mesoparasite from the mesopelagic environment of the region. This discovery highlights the unique biodiversity of the Red Sea and underscores the importance of exploring mesopelagic ecosystems.

## Abstract (fr)

Une nouvelle espèce du genre *Cardiodectes* Wilson, 1917 (Siphonostomatoida : Pennellidae), *Cardiodectes tofaili* n. sp., est décrite à partir de 13 femelles adultes issues de dix spécimens du poisson bioluminescent endémique *Vinciguerria mabahiss* (Stomiiformes : Phosichthyidae). Ces hôtes ont été capturés par inadvertance par un véhicule télécommandé à des profondeurs de 454 à 645 m dans les eaux pélagiques de la mer Rouge saoudienne. La nouvelle espèce est classée dans le groupe « *rubosus* », caractérisé par un tronc sans abdomen distinct. Elle se distingue de ses 12 congénères dans ce groupe par un cou court avec un quatrième somite pédigère distinct, et un tronc environ 5 fois plus long que large. L’analyse phylogénétique basée sur des séquences concaténées d’ADNr *18S* + *28S* confirme le caractère distinctif de la nouvelle espèce. Cette espèce est endémique de la mer Rouge et constitue le premier mésoparasite recensé dans l’environnement mésopélagique de la région. Cette découverte met en évidence la biodiversité unique de la mer Rouge et souligne l’importance d’explorer les écosystèmes mésopélagiques.

## Introduction

The Red Sea harbors a unique marine ecosystem characterized by its warm, deep-sea environment and exceptional biodiversity. Among its inhabitants, mesopelagic fish species, such as the endemic lightfish *Vinciguerria mabahiss* and the widely distributed skinnycheek lanternfish *Benthosema pterotum*, stand out as key components of this ecosystem [1, 20]. These species play crucial roles, not only as significant contributors to the vertebrate biomass of the mesopelagic zone [12, 19], but also through their dynamic interactions within coral reef habitats during their early development stages and ontogenetic vertical migrations [1, 20]. The ecological importance of these species includes their role in connecting the zooplankton food web with higher trophic levels, facilitating nutrient cycling within the pelagic environment and, through their extensive diel migrations [25], vertical trophic and biogeochemical connectivity between the upper and mesopelagic layers of the Red Sea.

Parasitism represents an important trophic link and a pervasive ecological strategy where one organism, the parasite, derives benefit from another, the host, often causing harm, while showcasing remarkable adaptations for survival [36]. Mesoparasites, in particular, exhibit specialized adaptations, with a significant portion of their body parts embedded within the host’s tissues [34]. Parasites vary widely in their host specificity, with some being generalists capable of infecting multiple host species, while others are specialists restricted to a single host species [51]. Understanding these parasitic relationships is crucial for comprehending the ecological dynamics of host populations and the role of these, often cryptic, components of food webs.

Among mesoparasites, copepods of the genus *Cardiodectes* Wilson, 1917, exemplify mesoparasitism within mesopelagic fish communities. Comprising 18 valid species divided into “*medusaeus*” and “*rubosus*” groups based on the presence or absence of a defined abdomen [4, 21], *Cardiodectes* species are parasitic across various teleost families (Table 1). Their life cycle often involves intermediate hosts such as molluscs and mesopelagic fish as definitive hosts [15, 32] – a phenomenon uncommon among fish-parasitic copepods. The evolutionary adaptations and host interactions of these copepods highlight their ecological significance and the complexity of parasitic life cycles.

**Table 1:** List of valid species of *Cardiodectes* Wilson C.B., 1917, including their geographic distribution and host fish species.

*Cardiodectes* species	Host family	Host species	Locality	Region	Reference(s)
*C. anchorellae* ^†^	Engraulidae	*Stolephorus tri*	India (Madras, Kerala)	Indian Ocean	[8, 13, 35]
	Engraulidae	*Thryssa hamiltonii*	Sri Lanka	Indian Ocean	[24, 35]
	Engraulidae	*Stolephorus indicus*	India (Madras, Kerala)	Indian Ocean	[13]
*C. bellottii* ^†^	Myctophidae	*Benthosema glaciale*	Mediterranean Sea	Mediterranean	[16, 22, 54]
	Myctophidae	*Ceratoscopelus townsendi*	Catalina Basin, California	Pacific Ocean	[32]
	Myctophidae	*Ceratoscopelus warmingii*	Eiao Island, Marquesas Archipelago	Pacific Ocean	[5]
	Myctophidae	*Diaphus rafinesquii*	Mediterranean Sea	Mediterranean	[22]
	Myctophidae	*Diaphus suborbitalis*	Japan (Misaki, Owase)	Pacific Ocean	[43, 54]
	Myctophidae	*Diaphus theta*	Catalina, Oregon Coast, San Clemente, Santa Barbara, Santa Cruz, San Pedro, Catalina Basins, California	Pacific Ocean	[30–32, 40]
	Myctophidae	*Gonichthys cocco*	Cape Point, South Africa; Off Mauritania, West Africa	Atlantic Ocean	[3, 32]
	Myctophidae	*Hygophum benoiti*	Mediterranean Sea	Mediterranean	[22]
	Myctophidae	*Lampadena dea*	Eiao Island, Marquesas Archipelago	Pacific Ocean	[5]
	Myctophidae	*Lampanyctodes hectoris*	South Africa (Cape Point, Saldanha Bay)	Atlantic Ocean	[3, 45]
	Myctophidae	*Lampanyctus ritteri*	Catalina Basin, San Clemente, San Pedro Basin, California	Pacific Ocean	[31, 32]
	Myctophidae	*Myctophum affine*	Mediterranean	Mediterranean	[7]
	Myctophidae	*Myctophum nitidulum*	East coast of Hamahiga Island, Japan	Pacific Ocean	[49]
	Myctophidae	*Myctophum* sp.	São Tiago Island, Cape Verde Islands	Atlantic Ocean	[9]
	Myctophidae	*Notoscopelus caudispinosus*	Genoa, Italy	Mediterranean	[6]
	Myctophidae	*Parvilux ingens*	Catalina Basin, California	Pacific Ocean	[32]
	Myctophidae	*Stenobrachius leucopsarus*	Los Angeles, Oregon Coast, San Clemente, San Diego, San Pedro, Santa Barbara, Santa Cruz, Catalina Basins, California	Pacific Ocean	[15, 29–32, 53, 54]
	Myctophidae	*Symbolophorus californiensis*	Catalina, San Pedro, San Clemente Basins, California	Pacific Ocean	[32]
	Myctophidae	*Tarletonbeania crenularis*	San Clemente, San Pedro, Catalina Basins, California	Pacific Ocean	[32, 40]
	Sternoptychidae	*Maurolicus muelleri*	Mauritius	Indian Ocean	[33]
*C. cristatus* ^†^	Myctophidae	*Diaphus suborbitalis*	Off the coast of Owase, Japan	Pacific Ocean	[43]
*C. frondosus* ^†^	Myctophidae	*Dasyscopelus spinosus*	Nuku Hiva, Marquesas Archipelago	Pacific Ocean	[41]
*C. longicervicus* ^†^	Myctophidae	*Dasyscopelus asper*	Off the coast of Owase, Japan	Pacific Ocean	[43]
*C. asper* ^§^	Gobiidae	*Trimma grammistes*	Izu-Oshima Island, Japan	Pacific Ocean	[50]
*C. bellwoodi* ^§^	Gobiidae	*Istigobius nigroocellatus*	Great Barrier Reef, Australia	Pacific Ocean	[49]
*C. bertrandi* ^§^	Gobiidae	*Eviota* sp.	Loyalty Islands, New Caledonia	Pacific Ocean	[50]
*C. boxshalli* ^§^	Scaridae	*Nicholsina usta*	Jamaica	Caribbean Sea	[4]
*C. hardenbergi* ^§^	Engraulidae	*Stolephorus* spp.	Java Sea	Indian Ocean	[28]
*C. krishnai* ^§^	Phosichthyidae	*Vinciguerria lucetia*	Arabian Sea	Arabian Sea	[35, 42]
*C. roatanensis* ^§^	Scaridae	*Nicholsina usta*	Roatan Island, Honduras	Caribbean Sea	[46]
*C. rotundicaudatus* ^§^	Gobiidae	*Suruga fundicola*	Sagami Bay, Japan	Pacific Ocean	[21]
*C. rubosus* ^§^	Apogonidae	*Apogon* sp.	Salomakië Island, Alaska	Pacific Ocean	[26]
	Dorosomatidae	*Harengula clupeola*	Cartagena, Columbia	Caribbean Sea	[52]
*C. shini* ^§^	Gobiidae	*Eviota* sp.	Okinawa-jima Island, Ryukyu Islands, Japan	Pacific Ocean, East China Sea	[14, 49]
	Gobiidae	*Eviota sebreei*			
	Gobiidae	*Pleurosicya micheli*			
	Gobiidae	*Priolepis* sp.			
*C. spiralis* ^§^	Anthiadinae	*Pseudanthias tuka*	Massas Island, Papua New Guinea	Pacific Ocean	[4]
*Cardiodectes* sp.	Pinguipedidae	*Parapercis sexfasciata*	Off the Tanegashima, Japan	Pacific Ocean	[57]
*C. vampire* ^§^	Chlorophthalmidae	*Chlorophthalmus corniger*	Arabian Sea	Arabian Sea	[2]
*C. tofaili* n. sp.^§^	Phosichthyidae	*Vinciguerria mabahiss*	Red Sea	Red Sea	Present study

^† “^*Medusaeus*” group, ^§^ “*Rubosus*” group.

Despite their global distribution in oceans, including the Atlantic [3, 9, 32, 45], Indian [8, 13, 24, 28, 33, 35], and Pacific Oceans [4, 5, 14, 15, 21, 26, 29-32, 40, 41, 43, 49, 50, 53, 54, 57], as well as the Mediterranean [6, 7, 16, 22, 54], Arabian [2, 35, 42], and Caribbean Seas [4, 46, 52], *Cardiodectes* species have notably not been previously documented in the Red Sea. This absence presents a gap in our understanding of the biogeography and diversity of mesoparasitic copepods and the ecology of mesopelagic fish in this unique marine environment.

Here we report the finding, during the Red Sea Decade Expedition (RSDE) 2022, of an undescribed *Cardiodectes* species parasitizing *Vinciguerria mabahiss*. This discovery not only fills a geographical gap in the distribution of *Cardiodectes*, but also presents an opportunity to elucidate its taxonomic identity and phylogenetic relationships within the family Pennellidae. By exploring these findings, this study aims to contribute to our understanding of mesoparasitic adaptations and their ecological implications in the mesopelagic zone of the Red Sea. Additionally, this discovery underscores the importance of ongoing exploration and research in uncovering the hidden biodiversity of mesopelagic ecosystems.

## Material and methods

### Ethics statement

All fish examined in this study were collected as incidental bycatch during remotely operated vehicle (ROV) operations conducted as part of the Red Sea Decade Expedition. The fish, attracted to the lights of the ROV, were impacted by the thruster motion during coring activities and were already dead at the time of collection; therefore, an Institutional Animal Care and Use Committee (IACUC) permit was not required. This research was conducted in accordance with ethical guidelines and was approved by the Institutional Biosafety and Bioethics Committee (IBEC) of the King Abdullah University of Science and Technology (approval number: 23IBEC055).

### Sample collection

Specimens of lightfish were bycatch from sediment core sampling during ROV dives conducted as part of the Red Sea Decade Expedition (RSDE) aboard R/V OceanXplorer between February and June 2022. The ROV entered mesopelagic fish aggregations near the seafloor, and some fish were displaced by thruster activity, settling on the seafloor. These fish were inadvertently trapped in sediment cores and processed onboard. All dives were categorized into different provinces based on the latitude defined by Raitsos and colleagues [37]. Upon collection, inspection of the fish revealed the presence of mesoparasitic pennellid copepods embedded in their bodies. Host specimens were stored at −20 °C onboard and transported to the laboratory at the King Abdullah University of Science and Technology (KAUST). Each fish was measured, weighed, and photographed using a Nikon D7500 digital camera equipped with a 105 mm Micro-Nikkor lens before preservation in 70% ethanol. Incidences of fish-parasite relationships were documented (Supplementary Table 1).

### Copepod preparation and examination and specimen deposition

Copepods were removed from the fixed fishes, soaked in lactophenol for approximately half a day, and dissected and examined using the wooden slide method [17]. Drawings were done with the aid of a drawing tube on an Olympus BX53 microscope, and terminology followed [18]. Copepod body parts were measured using an ocular micrometer, and measurements are reported in millimeters as a range. Body and cephalothorax lengths were measured from the tip of rostrum to the posterior tip of trunk and posterolateral lobes, respectively. Cephalothorax width was measured without branching processes. Type specimens examined in this study were deposited in the crustacean collection of the Senckenberg Research Institute (Frankfurt am Main, Germany). The fish specimens (*i.e.*, the hosts) were deposited in the ichthyological collection of the same museum.

### DNA extraction, amplification and sequencing

Genomic DNA for molecular analyses was extracted from a female paratype (AA10c) using a DNeasy Blood & Tissue kit (QIAGEN, Hilden, Germany), distinct from the holotype, SMF 63620. Two genes, *18S* rDNA and *28S* rDNA, were amplified using specific primer pairs: 18SU467F (5′–ATCCAAGGAAGGCAGCAGGC–3′) and 18SL1310R (5′–CTCCACCAACTAAGAACGGC–3′) [47], and 28SF (5′–ACAACTGTGATGCCCTTAG–3′) and 28SR (5′–TGGTCCGTGTTTCAAGACG–3′) [44] in a SimpliAmp™ thermal cycler (Applied Biosystems, Life Technologies, Waltham, MA, USA). PCR reactions were set up in a final volume of 25 μL, containing 1× QIAGEN^®^ Multiplex PCR master mix, 0.2 μM of both forward and reverse primers, 2 μL of genomic DNA, and RNase-free water to adjust the remaining volume. The thermal cycling profiles consisted of an initial denaturation at 95 °C for 15 min, followed by 35 cycles of denaturation at 94 °C for 30 s, annealing at 55 °C for 90 s, extension at 72 °C for 90 s, and a final elongation step at 72 °C for 10 min. PCR products were visualized on a 1.5% agarose gel (1× TAE) stained with SYBR Safe (Life Technologies) and electrophoresed at 110 V for 50 min. Amplicons were purified using Agencourt AMPure XP beads (Beckman Coulter, Brea, CA, USA) before bidirectional Sanger sequencing at the KAUST Bioscience Core Lab.

### Data analysis

Forward and reverse sequences generated from Sanger sequencing were assembled into contigs using Geneious Prime 2023.1.2 (https://www.geneious.com). Base call quality checks were performed manually prior to merging sequences. Merged sequences were aligned using the Geneious alignment tool before exporting for downstream analysis. Sequences were edited and trimmed to 650 bp (*18S*) and 541 bp (*28S*) and subsequently deposited in GenBank (Accession numbers: PQ108883 and PQ108885).

To infer phylogenetic relationships within the Pennellidae family, *18S* and *28S* reference sequences from 16 pennellid species were downloaded from GenBank and combined with our sequences, using *Caligus undulatus* as the outgroup (Supplementary Table 2). Sequences were concatenated into a 1,191 bp fragment (*18S* + *28S*), and alignments were exported to MEGA [48] to identify the best substitution model based on the lowest Bayesian Information Criterion (BIC) value. Three phylogenetic trees (*18S*, *28S*, *18S* + *28S*) were constructed in MrBayes using the mixed + gamma model with default parameters: mcmc ngen = 1,000,000, samplefreq = 500, printfreq = 500, diagnfreq = 5,000 [39]. The resulting .tre files were visualized in FigTree v1.3.1 [38] and edited in Inkscape. Parsimony network analysis was performed for two 18S sequences of *Cardiodectes* spp. using TCS networks implemented in PopArt [11].

The ecological assessment, including comparisons of standard length, wet weight, and infestation rates across localities, was conducted using RStudio (version 4.3.0). A Mann–Whitney U test was used to compare standard length and wet weight between non-infested and infested host fish. Differences in infestation rates among the five provinces (North, North-Central, Central, South-Central, and South) were analyzed using a Kruskal–Wallis test. The full R script is at available at: https://github.com/limkahkheng/mesopelagic-fish-parasite.

## Results

**Figure 1:**
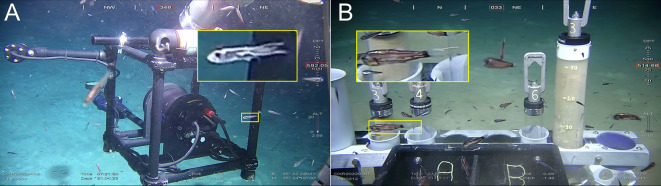
ROV footages of (A) lightfish (*Vinciguerria mabahiss*) and (B) lanternfish (*Benthosema* sp.) probably infested by mesoparasitic *Cardiodectes tofaili* n. sp.

During the Red Sea Decade Expedition 2022, two separate incidences of parasitic infection were observed in mesopelagic fish. Specifically, infestations were documented in one individual of *Vinciguerria mabahiss* (Fig. 1A) and one individual of *Benthosema* sp. (Fig. 1B) during separate ROV dives. A total of 158 host-parasite associations was observed among 368 dead specimens of *V. mabahiss* examined, equating to an infestation prevalence of 43% of the sampled populations. Fifteen fish individuals harbored two copepods, while the remaining fish each hosted a single parasitic copepod. The standard length (SL) of the host fish ranged from 14.89 to 27.75 mm, with an average of 19.97 mm ± 2.03 SD. The wet weight of the host fish varied between 0.03 and 0.17 g, averaging 0.08 g ± 0.03 SD.

**Figure 2:**
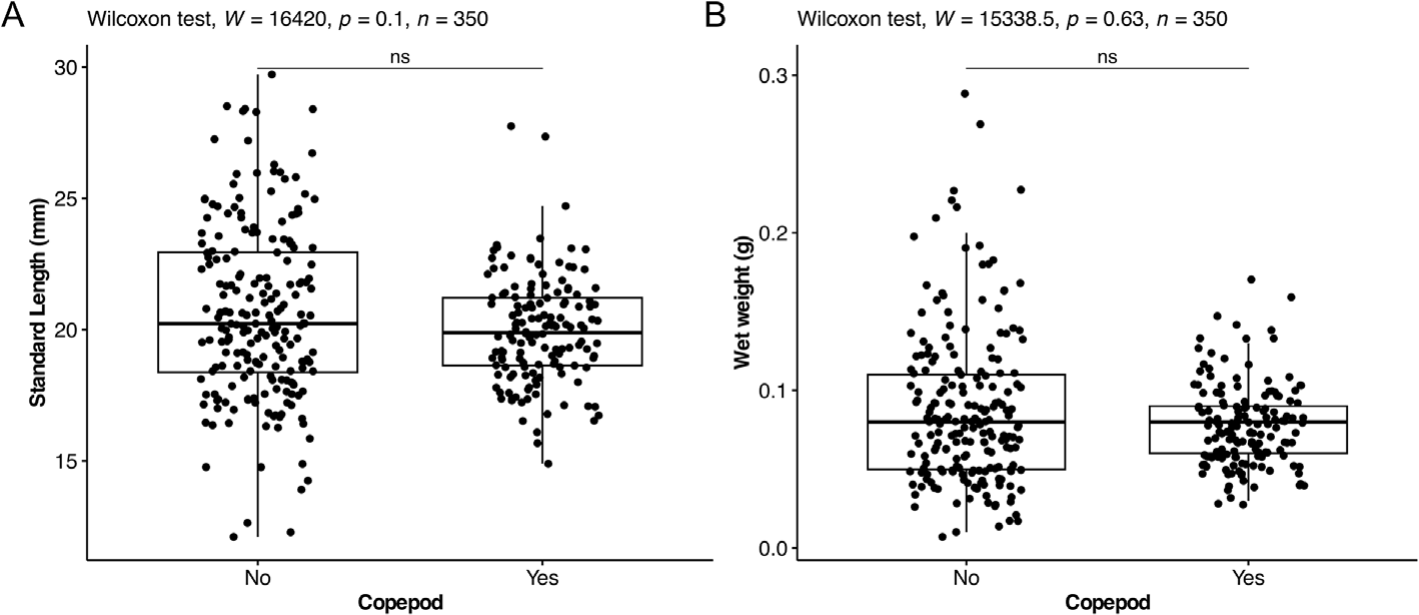
(A) Boxplot comparing the standard length of host fish infested by copepods versus non-infested. (B) Boxplot comparing the weight of host fish infested by copepods versus non-infested. “ns” denotes non-significant.

The Mann–Whitney U test indicated no significant difference in standard length between non-infested (*N* = 204, median = 20.2 mm) and infested (*N* = 146, median = 19.9 mm) host fish (*W* = 16420, *p* = 0.1, Fig. 2A). Similarly, there was no significant difference in wet weight between non-infested and infested host fish (*N* = 350, median = 0.08 g) (*W* = 15338, *p* = 0.63, Fig. 2B). The Kruskal–Wallis test showed no significant differences in the mean ranks of infestation rates among the five provinces (*N* = 350, *H* (4) = 1.9757, *p* = 0.7402).

### Taxonomy

Order Siphonostomatoida Burmeister, 1835

Family Pennellidae Burmeister, 1835

Genus *Cardiodectes* Wilson, 1917

### *Cardiodectes tofaili* n. sp. (Figs. 3–5)

urn:lsid:zoobank.org:act:3EB69733-630D-476D-A3FF-AEDF41240785

**Figure 3:**
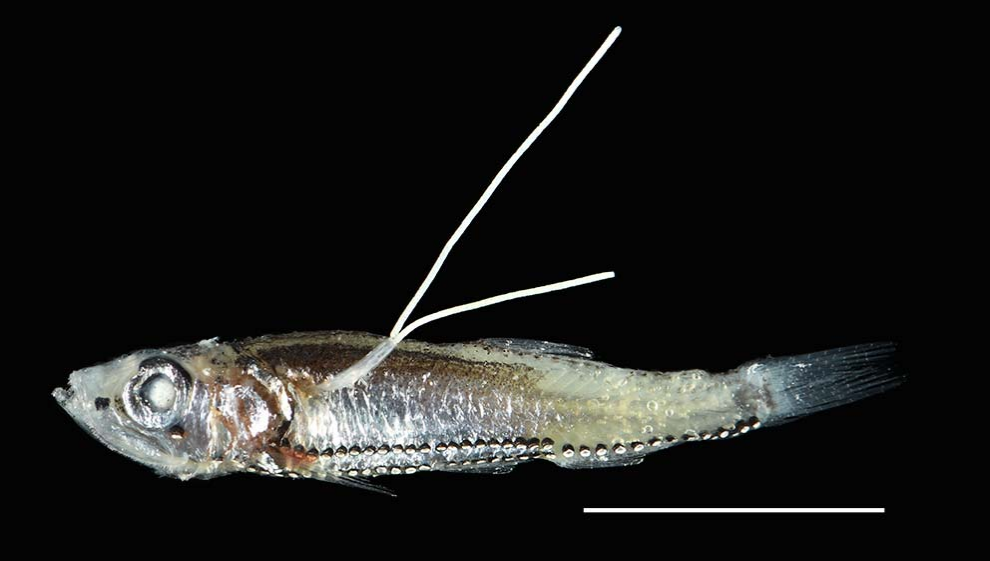
A specimen of *Vinciguerria mabahiss* infected by an adult female of *Cardiodectes tofaili* n. sp. carrying a pair of egg strings. Scale bar 10 mm.

Type-host: *Vinciguerria mabahiss* Johnson & Feltes, 1984 (Stomiiformes: Phosichthyidae) (SMF 40114).

Type-locality: CHR0269 (23°47′56.1″N, 38°12′10.3″E), off Yanbu, Red Sea, Saudi Arabia, 645 m depth, 9 May 2022.

Other localities: CHR0197 (21°54′16.2″N, 38°45′28.5″E), off Jeddah, Red Sea, Saudi Arabia, 642 m depth, 22 February 2022; CHR0198 (21°38′25.3″N, 38°54′46.9″E), off Jeddah, Red Sea, Saudi Arabia, 612 m depth, 23 February 2022; CHR0199 (21°25′31.8″N, 39°01′50.2″E), off Jeddah, Red Sea, Saudi Arabia, 454 m depth, 24 February 2022; CHR0201 (20°38′09.2″N, 39°28′03.4″E), off Jeddah, Red Sea, Saudi Arabia, 564 m depth, 26 February 2022.

Attachment site: all type specimens were attached to the specimens of *V. mabahiss*. The cephalothorax and neck region of the copepod were embedded in the musculature of the host’s trunk, while remaining portion, *e.g.*, trunk and egg strings, exposed outside (Fig. 3).

Type-material: holotype female (SMF 63620) and paratypes: 12 females (SMF 63621-63629).

Prevalence and intensity: one to two copepods in a single host.

Etymology: the species name *tofaili* is derived from the Arabic word for “parasite,” honoring the nation’s commitment to deep-sea exploration in the Red Sea.

#### Description (Figs. 4–5; Table 2)

**Figure 4:**
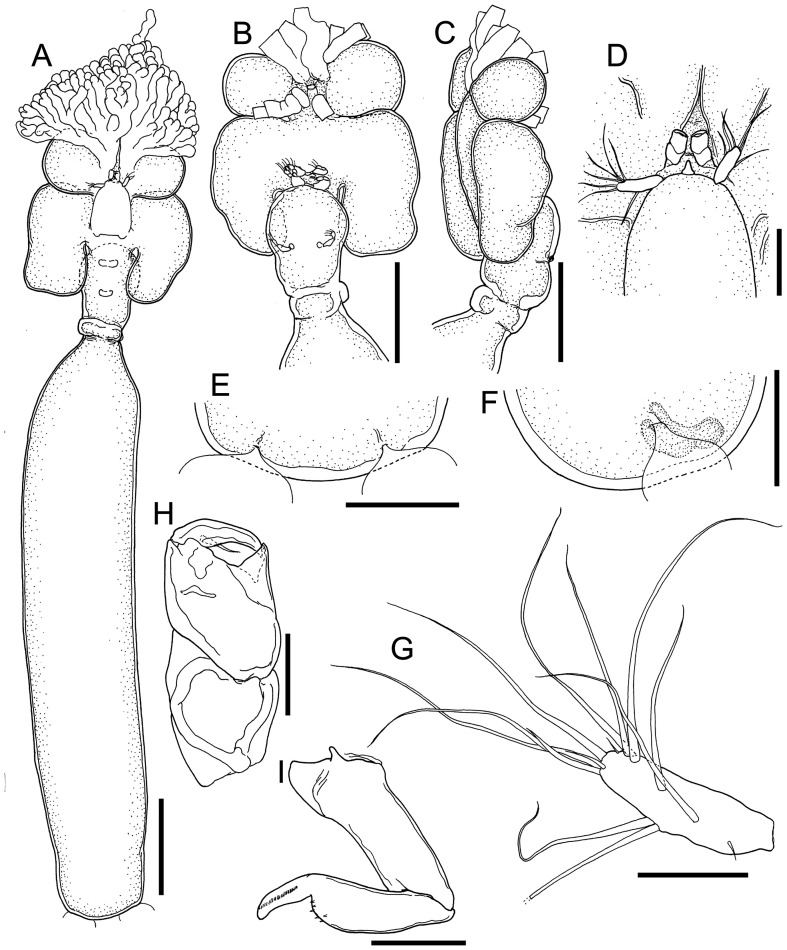
*Cardiodectes tofaili* n. sp., postmetamorphic adult female, Holotype SMF 63620. A, habitus, dorsal; B, cephalothorax and neck region with digitiform processes removed, ventral; C, same, right side, lateral; D, anterior part of cephalothorax with antennules, antennae and rostrum; E, posterior portion of trunk, ventral; F, same, right side, lateral; G, left antennule, anterior; H, left antenna, anterior; I, left maxilla, anterior. Scale bars: A, 500 μm; B, C, 400 μm; D, 100 μm; E, F, 20 μm; G, 40 μm; H, I, 20 μm.

Postmetamorphic adult female. Body (Fig. 4A) 4.71 mm long, comprising cephalothorax, neck region and trunk. Cephalothorax (Figs. 4A–4D) almost as long as wide or slightly longer than wide, 0.86 × 0.85, bearing pairs of nodular and branching anterior processes on anterodistal portion of cephalothorax and pair of anterior spherical lobes, as well as pair of rounded posterolateral lobes extending backwards. Neck region (Figs. 4A–4C) narrow, 0.49 × 0.34, straight: second pedigerous somite swollen, wider than third pedigerous somite; fourth pedigerous somite distinctly segmented and slightly expanded laterally. Trunk (Fig. 4A) slender, *ca.* five times as long as wide, 3.01 × 0.58, with round posterior margin. Egg strings straight (Fig. 3) and uniseriate, originating at posterolateral genital apertures.

**Figure 5:**
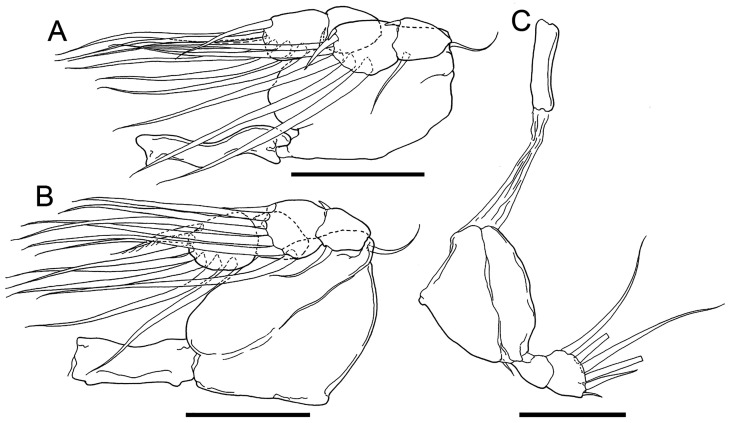
*Cardiodectes tofaili* n. sp., postmetamorphic adult female, Holotype SMF 63620. A, left leg 1 with intercoxal sclerite, posterior; B, left leg 2 with intercoxal sclerite, posterior; C, right leg 3 with intercoxal sclerite, anterior. Scale bars: A–C, 40 μm.

**Table 2:** Armature of legs 1 to 3 of adult female of *Cardiodectes tofaili* n. sp.

	Protopod	Exopod	Endopod
Leg 1	1-0	1-1; 7	0-0; 7
Leg 2	1-0	1-1; 7	0-0; 7
Leg 3	1-0	0-0; 6	Absent

Rostrum, antennules, and antennae situated closely to each other on anterodorsal portion of cephalothorax (Fig. 4D). Rostrum convex with rounded free margin (Figs. 4A, 4E, 4F). Antennule (Fig. 4G) unsegmented, rod-like, bearing at least 5 setae mainly on anterior margin; distal tip bearing at least 8 setae. Antenna (Fig. 4H) 3-segmented, chelate, typical pennellid in form; proximal segment unarmed; middle segment bearing inner pointed projection and pocket; terminal claw with small basal seta on posterior surface. Mouth tube and maxilla situated on anterior part of ventral surface of cephalothorax. Maxilla (Fig. 4I) 2-segmented: proximal segment unarmed; terminal segment separated two parts by constriction at distal four third, bearing fine spinules on basal part and a row of fine spinules on distal claw. Maxilliped absent.

Legs 1 and 2 (Figs. 5A, 5B) biramous, situated on posterior part of cephalothorax (Fig. 4B). Leg 3 (Fig. 5C) uniramous, situated behind anterior swollen on neck. Armature formula of all three legs shown in Table 2. Protopod of leg 3 (Fig. 5C) separated from intercoxal sclerite by long gap.

*Variability of female morphology*. The morphology of the female paratypes is as in the holotype. The measurements of the paratypes are as follows: body length 3.21–4.46; cephalothorax length 0.65–0.88; cephalothorax width 0.50–0.74; neck length 0.43–0.53; neck width 0.37; trunk length 2.25–2.92; trunk width 0.45–0.60; trunk 4.87 to 4.95 times longer than wide (*n* = 2).

*Remarks*. *Cardiodectes tofaili* n. sp. shares the characteristic of having a trunk without an abdomen with 12 other congeners in the “*rubosus*” group (see [4, 21]). Although the trunk length of the new species is *ca.* 5 times longer than wide and only *C. krishnai* Sebastian, 1968 has such elongate trunk (*i.e.*, more than five times longer than wide), among the 12 congeners (see [2, 4, 21, 26, 28, 42, 46, 49, 50]). The new species is similar to *C. krishnai* but differs by having the short neck region (*i.e.*, the trunk 5.2 to 6.1 times longer than the neck region) with the second pedigerous somite swollen and the free fourth pedigerous somite (*vs.* the trunk 3 times longer than the neck region with the second pedigerous somite not swollen and the fourth pedigerous somite not clearly segmented, Sebastian, 1968, fig 1).

### Phylogeny of Pennellidae

**Figure 6:**
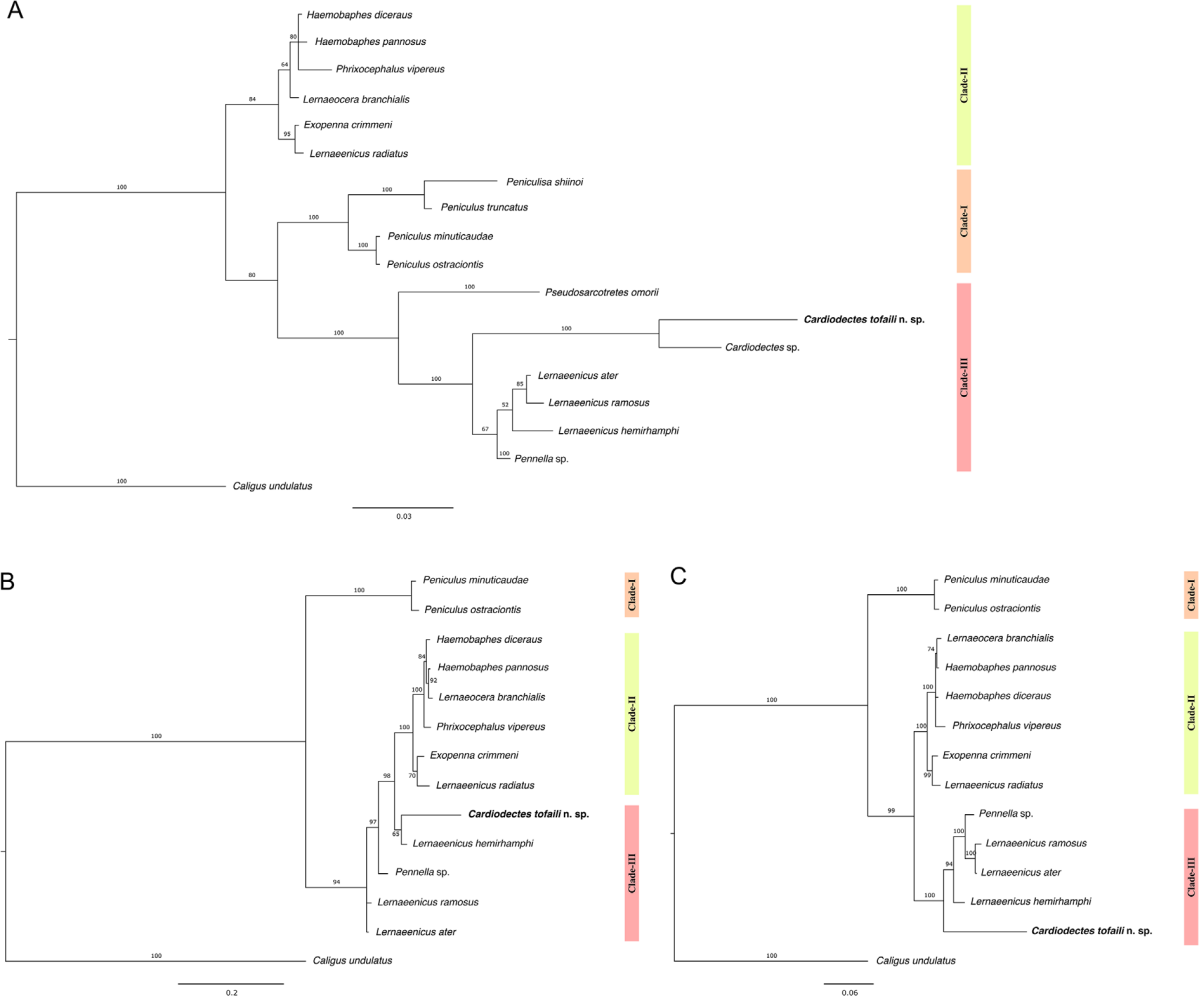
Phylogenetic trees of pennellids based on (A) *18S* rDNA, (B) *28S* rDNA, and (C) concatenated *18S* and *28S* rDNA sequences, using *Caligus undulatus* as the outgroup taxa. Node numbers indicate bootstrap values for analyses of posterior probabilities (%) for Bayesian analysis. Scale bars represent nucleotide changes per site.

**Supplementary Figure S1.**
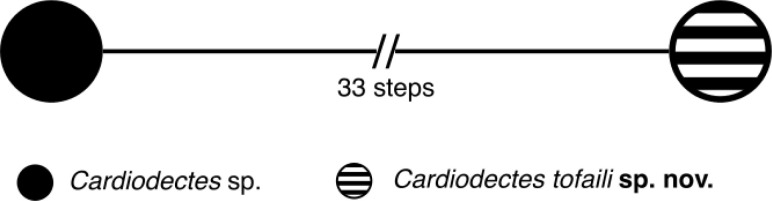
Statistical parsimony network of *Cardiodectes* based on *18S* rDNA sequences. The network illustrates the genetic differentiation between the newly described Red Sea species, *Cardiodectes tofaili* n. sp., and the Japanese species, *Cardiodectes* sp., with the latter being the only sequence available from this genus in GenBank (LC777455). Each circle represents a haplotype, with species distinguished by a different pattern.

Analysis of *18S* (650 bp), *28S* (541 bp) and concatenated *18S* + *28S* (1,191 bp) sequences from 13–17 species recovered three clades as proposed by [56] (Figs. 6A–6C). Clade-I consisted of *Peniculisa* and *Peniculus* spp., while Clade-II consisted of *Haemobaphes* spp., *Phrixocephalus vipereus, Lernaeocera branchialis*, *Exopenna crimmeni*, and *Lernaeenicus radiatus*. Clade-III consisted of *Pseudosarcotretes omorii*, *Cardiodectes* spp. including the Red Sea species, *Lernaeenicus* spp., and *Pennella* sp. The Red Sea species (*Cardiodectes tofaili* n. sp.) is genetically differentiated from the Japanese species (*Cardiodectes* sp.) by 33 mutational steps based on TCS network analysis of 18S rDNA sequences (Supplementary Figure S1). Unfortunately, the genetic sequence for *Cardiodectes krishnai*, which is geographically closer and potentially more closely related to *Cardiodectes tofaili* n. sp. than *Cardiodectes* sp., is not available, limiting direct comparison and highlighting the need for further genetic characterization of this genus.

## Discussion

This study contributes to our understanding of the taxonomy and phylogeny of the Pennellidae family through the discovery of a new species, *Cardiodectes tofaili* n. sp., parasitizing the endemic *Vinciguerria mabahiss* in the Red Sea. By incorporating the Red Sea species into our analysis, we conducted a revision of the pennellid phylogenetic tree using *18S* rDNA gene sequences from 16 taxa, including both identified species and representative genera. This allowed for a re-evaluation of previous findings by [56, 57]. Our phylogenetic analysis, using *Caligus undulatus* as the outgroup, clearly distinguishes the Red Sea species from other pennellids. We successfully recovered the three clades proposed by [56], placing *Cardiodectes tofaili* n. sp. in Clade-III alongside *Pseudosarcotretes omorii*, *Cardiodectes* spp., *Lernaeenicus* spp., and *Pennella* sp. with a high posterior probability (PP = 100%) (Fig. 6A). The assignment was supported by phylogenetic tress constructed using *28S* and concatenated *18S* + *28S* rDNA gene sequences (Figs. 6B, 6C).

The placement of *Cardiodectes tofaili* n. sp. within Clade-III suggests a shared evolutionary history with its sister taxa, characterized by adaptations such as the cephalothoracic holdfast, facilitating attachment and survival in the mesopelagic environment [57]. Several species within Clade-III are known to parasitize small, deep-sea fish such as cardinal fish, lanternfish, and lightfish [5, 43, 55, 57]. The relatively large size of these mesoparasitic copepods compared to their small hosts suggests specialized adaptations beyond occupying open spaces like gills and mouths [57]. As proposed by Yumura *et al.* [56], pennellids may have expanded their host range by adopting mesoparasitic infection strategies. For instance, the cephalothoracic holdfast of *Cardiodectes tofaili* n. sp. appears to facilitate penetration and anchoring to host muscle tissue, particularly in small, fast-swimming hosts like *Vinciguerria mabahiss* [23]. Our observation of a similar parasite on a lanternfish *Benthosema* sp. from ROV footage suggests a potential broader host range. However, definite evidence of taxonomic affinity awaits further investigation, as no parasites were found on collected *Benthosema* sp. specimens, indicating that although possible, the presence of *Cardiodectes* seems very rare. Our results suggest that *Cardiodectes tofaili* n. sp. primarily infects the phosichthyids, whereas Yumura and colleagues reported a *Cardiodectes* species infecting the grub fish *Parapercis sexfasciata* in the Pacific Ocean [57]. This discrepancy suggests that *Cardiodectes* may infect both midwater fish and demersal fish. However, our observation from ROV footage hints a potential, albeit lower, infestation rate of myctophids (*Benthosema* sp.), with no fish-parasite events detected in 123 dead specimens of *Benthosema* sp. examined.

Further studies are warranted to explore the ecological implications of these parasitic relationships and their potential impacts on host fitness and population dynamics within the Red Sea ecosystem. In our study, *Cardiodectes tofaili* n. sp. was found on a wide range of host sizes, ranging from 14.89 to 27.75 mm SL. Importantly, these infestations occurred irrespective of the size of the host, predominantly in the region behind the operculum. Multiple infestations were noted and appeared independent of host sizes. Mesopelagic fish typically live for one to five years [10], during which parasite infections can be transmitted through host behaviors and dietary habits. The life cycle of *Cardiodectes* typically involves two hosts, with pelagic gastropods acting as intermediate hosts and mesopelagic fish as definitive hosts [32]. Pelagic gastropods, particularly of the genera *Clio* such as *Clio pyramida* and *C. balantium*, as well as the heteropod *Carinaria japonica*, are known intermediate hosts of *Cardiodectes* infecting myctophids [32]. Mesopelagic fish in the Red Sea, including *V. mabahiss*, are known to prey on gastropods during their diel vertical migrations [27], which likely contributes to the observed infestation patterns of *Cardiodectes tofaili* n. sp. Further investigations are needed to verify the species identity of intermediate hosts of *Cardiodectes* in the Red Sea.

## Supplementary materials

The supplementary material of this article is available at https://www.parasite-journal.org/10.1051/parasite/2025038/olm.

*Supplementary Table 1*. Metadata on the collection of the host *Vinciguerria mabahiss* infected by the mesoparasitic *Cardiodectes tofaili* sp. nov., during the Red Sea Decade Expedition (RSDE) 2022. Data are displayed for complete individuals with morphometric measurements. Twelve incomplete host fish infected with copepods are not shown as measurements could not be obtained. *Supplementary Table 2*. *18S* and *28S* sequences of pennellids and caligid (*Caligus undulatus*) used in current study, with GenBank accession numbers.
