# Supplementary material for: Discovery of new species of mesoparasitic pennellid (Copepoda: Siphonostomatoida) from the endemic mesopelagic lightfish Vinciguerria mabahiss in the Red Sea
Source: Parasite. 2025 Jul 16;32:43. doi: 10.1051/parasite/2025038 (PMC12266664; doi:10.1051/parasite/2025038)
Supplement: Supplementary file 1 — Supplementary Table 1. Metadata on the collection of the host Vinciguerria mabahiss infected by the mesoparasitic Cardiodectes tofaili sp. nov., during the Red Sea Decade Expedition (RSDE) 2022. Data are displayed for complete individuals with morphometric measurements. Twelve incomplete host fish infected with copepods are not shown as measurements could not be obtained. [file parasite-32-43-s1.pdf]

Supplementary Table 1. Metadata on the collection of the host *Vinciguerria mabahiss* infected by the mesoparasitic *Cardiodectes tofaili* **sp. nov.**, during the Red Sea Decade Expedition (RSDE) 2022. Data are displayed for complete individuals with morphometric measurements. Twelve incomplete host fish infected with copepods are not shown as measurements could not be obtained.

**Province**	**Latitude**	**Longitude**	**Depth (m)**	**Dive**	**No. of host fish**	**Copepod Frequency**	**Host Wet Weight (g)**				**Host Standard Length (mm)**			
							**Min**	**Max**	**Mean**	**SD**	**Min**	**Max**	**Mean**	**SD**
S-Central	20.180538	39.306439	526	CHR0188	1	1	-	-	0.08	-	-	-	21.33	-
Central	21.904512	38.757906	642	CHR0197	91	1	0.03	0.16	0.08	0.02	14.89	23.14	19.59	1.70
					12	2	0.05	0.13	0.08	0.02	17.54	22.73	20.06	1.52
Central	21.640355	38.913025	612	CHR0198	2	1	0.04	0.10	0.07	0.05	17.06	22.40	19.73	3.78
Central	21.425488	39.030606	454	CHR0199	19	1	0.03	0.17	0.08	0.04	16.09	27.75	20.59	3.26
S-Central	20.635875	39.467614	564	CHR0201	4	1	0.07	0.10	0.08	0.02	18.80	22.69	20.71	1.65
					1	1	-	-	0.08	-	-	-	20.87	-
S-Central	20.24747	39.508776	514	CHR0202	1	1	-	-	0.04	-	-	-	17.23	-
S-Central	19.856452	40.126386	497	CHR0203	1	1	-	-	0.09	-	-	-	21.59	-
Central	22.484302	38.865877	618	CHR0265	1	1	-	-	0.12	-	-	-	22.12	-
N-Central	23.798929	38.202857	645	CHR0269	9	1	0.06	0.11	0.08	0.01	18.63	23.23	20.95	1.52
					2	2	0.03	0.08	0.05	0.03	18.58	22.82	20.70	3.00
N-Central	25.768232	36.514866	599	CHR0275	2	1	0.05	0.14	0.09	0.07	20.12	24.71	22.42	3.25
				**Overall**	**146**	**-**	**0.03**	**0.17**	**0.08**	**0.03**	**14.89**	**27.75**	**19.97**	**2.03**
