# Supplementary material for: Discovery of new species of mesoparasitic pennellid (Copepoda: Siphonostomatoida) from the endemic mesopelagic lightfish Vinciguerria mabahiss in the Red Sea
Source: Parasite. 2025 Jul 16;32:43. doi: 10.1051/parasite/2025038 (PMC12266664; doi:10.1051/parasite/2025038)
Supplement: Supplementary file 2 — Supplementary Table 2. 18S and 28S sequences of pennellids and caligid (Caligus undulatus) used in current study, with GenBank accession numbers. [file parasite-32-43-s2.pdf]

Supplementary Table 2. *18S* and *28S* sequences of pennellids and caligid (*Caligus undulatus*) used in current study, with GenBank accession numbers.

Species	GenBank Accession no.		References
	28S	***18S***	
Peniculus minuticaudae Shiino, 1956	LC586450	LC586436	[1]
Peniculus ostraciontis Yamaguti, 1939	LC586449	LC586437	[1]
Haemobaphes diceraus Wilson C.B., 1917	LC586447	LC586435	[1]
Haemobaphes pannosus Kabata, 1979	KR048859	KR048773	[55]
Lernaeocera branchialis Linnaeus, 1767	MN520223	AY627030	[3, 4]
Phrixocephalus viperous Shiino, 1956	LC650962	LC650961	[1]
Exopenna crimmeni Boxshall, 1986	LC586448	LC586434	[1]
Lernaeenicus radiatus Lesueur, 1824	MN520221	MN523342	[4]
Lernaeenicus hemirhamphi Kirtisinghe, 1932	LC586446	LC586441	[1]
Pennella sp. Oken, 1815	LC586445	LC586438	[1]
Lernaeenicus ramosus Kirtisinghe, 1956	LC586444	LC586439	[1]
Lernaeenicus ater Shiino, 1958	LC586443	LC586440	[1]
Cardiodectes tofaili **sp. nov.**	PQ108883	PQ108885	Current study
Peniculisa shiinoi Izawa, 1965	NA	LC777454	[5]
Peniculus truncates Shiino, 1956	NA	LC777453	[5]
Cardiodectes sp. Wilson C.B., 1917	NA	LC777455	[5]
Pseudosarcotretes omorii Yumura, Nishikawa & Ohtsuka, 2024	NA	LC777456	[5]
Caligus undulatus Shen & Li, 1959	LC586451	LC586442	[1]

NA, not available
